# Evaluation of the Alterations in Central Cholinergic Neurotransmission in Aging and Amyloid Precursor Protein Knock‐In Mice

**DOI:** 10.1111/jnc.70081

**Published:** 2025-05-13

**Authors:** Itsumi Nagai‐Arakawa, Ikunobu Muramatsu, Junsuke Uwada, Yo Tsuda, Akinori Tokunaga, Ai Irie, Hideyuki Maeda, Yuta Madokoro, Toyohiro Sato, Yuto Uchida, Takashi Saito, Takaomi C. Saido, Kiyonao Sada, Takayoshi Masuoka, Noriyuki Matsukawa

**Affiliations:** ^1^ Department of Neurology Nagoya City University Graduate School of Medicine Nagoya Aichi Japan; ^2^ Department of Genomic Science and Microbiology Faculty of Medical Sciences, University of Fukui Fukui Japan; ^3^ Department of Pharmacology School of Medicine, Kanazawa Medical University Uchinada Ishikawa Japan; ^4^ Division of Laboratory Animal Resources Life Science Support Center, University of Fukui Fukui Japan; ^5^ Department of Neurocognitive Science Institute of Brain Science, Nagoya City University Graduate School of Medical Sciences Nagoya Aichi Japan; ^6^ Laboratory for Proteolytic Neuroscience RIKEN Center for Brain Science Saitama Japan

**Keywords:** acetylcholine, aging, Alzheimer's disease, cholinergic

## Abstract

A progressive decline in cognitive function occurs as a result of aging and Alzheimer's disease (AD) and is primarily associated with diminished cholinergic neurotransmission. However, the precise mechanisms contributing to cholinergic dysfunction are not fully elucidated. Herein, we evaluated the cholinergic system in wild type (WT) mice and AD‐model (*App* ^*NL‐G‐F*^) mice exhibiting overproduction of amyloid‐beta 42 (Aβ_42_). In superfusion experiments, [^3^H]acetylcholine (ACh) release from the frontal cortex and hippocampal segments preloaded with [^3^H]choline exhibited no significant differences between adult (6–8 months old) and aged (12–17 months old) WT mice. Uptake of [^3^H]choline via the high‐affinity choline transporter 1 (CHT1) and the subsequent formation/storage of [^3^H]ACh showed a moderate tendency to decrease associated with aging. In contrast, in *App* ^*NL‐G‐F*^ mice, [^3^H]ACh release was significantly reduced in both the adult and aged groups, with reductions closely related to impaired CHT1 activity and diminished ACh synthesis/storage at cholinergic terminals. Presynaptic cholinergic feedback mechanisms regulating ACh release, as well as the density and subtype distribution of muscarinic ACh receptors, were minimally affected by both aging and Aβ_42_ overproduction. These results support the Aβ hypothesis, suggesting that presynaptic cholinergic dysfunction arises early and is specifically caused by decreased CHT1 function in the AD forebrain, independent of age‐dependent degeneration.

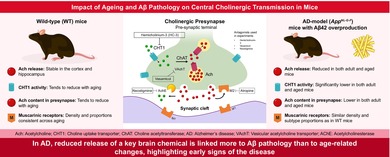

## Graphical abstract

In Alzheimer's disease, reduced release of ACh is linked more to Aβ pathology than to age‐related change, highlighting an early sign of disease.

**Abbreviations**
[^3^H]QNB: [^3^H]quinuclidinyl benzilate
ACh: acetylcholine
AChE: acetylcholine esterase
aCSF: artificial cerebrospinal fluid
AD: Alzheimer's disease
APP: Amyloid precursor protein
*App* ^*NL‐G‐F*^ mice: Alzheimer's disease model mice
ChAT: choline acetyltransferase
CHT1: high‐affinity choline uptake transporter
HC‐3: hemicholinium‐3
HPLC: high‐performance liquid chromatography
VAChT: vesicular acetylcholine transporter
WT: wild type

## 1 Introduction

Dementia is a pressing social issue that requires urgent attention, particularly in developed countries. Alzheimer's disease (AD) is the leading cause of dementia, accounting for approximately 60% of cases. AD typically manifests as a progressive decline in cognitive function and daily activities (Li et al. 2022; Lian et al. 2019). The pathological changes in AD include the deposition of amyloid β (Aβ), senile plaques, and phosphorylated tau protein, as well as the intracellular formation of neurofibrillary tangles (NFTs), as described by the amyloid cascade hypothesis (Kepp et al. 2023; Neve and Robakis 1998; Rischel et al. 2023). Consequently, murine models exhibiting Aβ_42_ overproduction have been extensively utilized in AD research (Apelt et al. 2002; Saito et al. 2014). Recently, the anti‐amyloid antibodies Lecanemab and Donanemab have been introduced as disease‐modifying therapies in clinical settings (Klein et al. 2024; van Dyck et al. 2023).

Acetylcholine (ACh) is widely distributed in the central nervous system and has been implicated to play a critical role in modulating cognitive performance, learning and memory processes, as well as in attention and other higher‐order brain functions (Everitt and Robbins 1997; Hampel et al. 2018; Sarter and Parikh 2005; Wess et al. 2007). During aging, the cholinergic system has been assumed to undergo moderate degenerative changes, resulting in cholinergic hypofunction, which is related to progressive memory deficits (Dumas and Newhouse 2011; Mesulam 2004; Schliebs and Arendt 2011). In AD, a main pathological change is cholinergic dysfunction without cholinergic neuronal degeneration, which occurs early. This is followed by a severe loss of cholinergic innervation, which has been extensively documented in the advanced stages of the disease (Blennow et al. 2006; Coyle et al. 1983; Haam and Yakel 2017; Hampel et al. 2018; Nordberg 1992; Reinikainen et al. 1988). This key pathological change is elucidated by the cholinergic hypothesis (Bartus et al. 1982; Hampel et al. 2018). Cholinesterase inhibitors that enhance cholinergic function have been employed in an attempt to ameliorate clinical symptoms associated with AD (Zuliani et al. 2024). Despite this, the precise mechanisms underlying the decrease in ACh release and choline metabolism remain poorly understood.

Changes in cholinergic function during aging and AD have been widely documented using various cholinergic parameters, such as high‐affinity choline uptake transporter (CHT1), vesicular ACh transporter (VAChT), ACh esterase (AChE), muscarinic and nicotinic ACh receptors, ACh quantity, release, and neuronal density (Apelt et al. 2002; Liu et al. 2022). Among these parameters, ACh release provides the most direct evidence of changes in cholinergic transmission. ACh release is known to be regulated by various factors, including the activities of CHT1 and VAChT, as well as presynaptic feedback through cholinergic and dopaminergic receptors (Zhang et al. 2002; Garcao et al. 2013, 2014; Muramatsu et al. 2022; Arakawa et al. 2023). The requirement for simple yet precise measurement of ACh release has become apparent.

Recently, we developed an ultra‐mini superfusion system that can directly measure [^3^H]ACh release from [^3^H]choline‐preloaded cholinergic neurons/terminals in vitro, revealing some pharmacological characteristics of cholinergic transmission (Arakawa et al. 2023; Muramatsu et al. 2017, 2019). In the present study, we applied the ultra‐mini superfusion technique to small segments of mouse brain tissues (frontal cortex, hippocampus and striatum) and compared the effects of aging and Aβ_42_ overproduction on cholinergic transmission. In this study, we used *App* ^*NL‐G‐F*^ mice as an AD model and compared them with age‐matched wild type (WT) mice (adult: 6–8 months old, aged: 12–17 months). The *App* ^*NL‐G‐F*^ mice are knock‐in models harboring the Swedish (KM670/671NL) and Beyreuther/Iberian (I716F) mutations with the Arctic (E693G) mutation in the Amyloid precursor protein (APP) gene, leading to Aβ_42_ overproduction. These mice exhibit more typical Aβ pathology, neuroinflammation, and memory impairment in an age‐dependent manner when compared to previously reported AD‐model mice (Saito et al. 2014).

## 2 Materials and Methods

### 2.1 Animals

All animal experiments were approved by the ethics committees of Nagoya City University Graduate School of Medical Sciences (permit No. 18‐151), the University of Fukui (approval No. R04018), and Kanazawa Medical University (approval No. 2020‐26). These experiments conformed to the guidelines for the use of laboratory animals published by the Japanese government (Law No. 105, October 1973). The AD‐model mice used were *App* ^*NL‐G‐F*^ mice, which had been generated using C57BL/6 mouse (Japan SCL Inc., Hamamatsu, Japan) by co‐workers (T.S., T.C.S) (Saito et al. 2014) and offered from RIKEN Center for Brain Science, (Wako, Japan). The AD mice exhibit typical Aβ pathology, neuroinflammation, and memory impairment. Wild type (WT), age‐matched, non‐knock‐in littermates served as controls. Three or four mice were housed in a clear acrylic cage and maintained under controlled temperature (24°C) and humidity (45%) conditions, with a 12‐h light/dark cycle (lights on 08:00 to 20:00) and free access to food and water. Mice of both sexes were included in this study (Table S1). Adult mice were defined as 6–8 months old, and aged mice as 12–17 months old. Mice were anesthetized with isoflurane (4% in air) and sacrificed by cervical dislocation. The brain was rapidly isolated and immersed in standard artificial cerebrospinal fluid (aCSF) consisting of 124 mM NaCl, 3 mM KCl, 1.2 mM MgCl_2_, 2.4 mM CaCl_2_, 1.2 mM NaH_2_PO_4_, 26 mM NaHCO_3_, and 10 mM D‐(+)‐glucose (pH 7.4). This solution was oxygenated with a mixture of 95% O_2_ and 5% CO_2_ beforehand and maintained at 0°C (Muramatsu et al. 2022). The frontal cortex, hippocampus, and striatum were carefully cut into small pieces at 4°C using a razor and fine ophthalmic scissors under a stereoscopic microscope. Segments prepared from each brain area were randomly assigned for three or more experiments. The sample size (*n* = 4–6) was determined according to previous studies (Arakawa et al. 2023; Muramatsu et al. 2022).

### 2.2 Superfusion Experiments

Hippocampal, frontal cortical, and striatal segments were used in the superfusion experiments. Due to their small size (approximately 1 mm in length, 0.5–1 mm in width, and 0.2–0.3 mm in thickness), an ultra‐mini vessel (vessel type B, Muramatsu et al. 2024a) was developed. Briefly, this vessel is a small tube (2.4 mm in internal diameter, 12 mm in length) with a volume of less than 50 μL. Two distinct platinum electrodes were placed at the top and bottom of the mini‐vessel for electrical stimulation. After incubation with 0.1 μM [^3^H]choline for 15 min, four segments were floated in the vessel and then set in the superfusion apparatus. The vessel was constantly perfused with oxygenated aCSF at a flow rate of approximately 0.8 mL/min using a peristaltic pump (Arakawa et al. 2023; Muramatsu et al. 2017). Electrical stimulation was delivered using an electronic stimulator with a current booster (SEN‐3401MG; Miyuki‐Giken, Tokyo, Japan). The parameters of the electrical pulses were 300 μs in duration and 26 V for the voltage. The superfusion apparatus, except for the stimulator and collecting vials, was kept at 37°C. The superfused medium was collected every minute, and radioactivity was measured. The first electrical stimulation (S1) was applied 40 min after superfusion commenced, and the second stimulation (S2) was applied at 55 min. The net increase in [3H]efflux induced by electrical stimulation was calculated by subtracting the average of three fractions (the expected basal value) preceding each stimulation. The net [3H]efflux provoked by electrical stimulation exhibited reproducibility between S1 and S2, in the absence of any treatments. To examine presynaptic modulation of ACh release via muscarinic and nicotinic receptors, either atropine (1 μM) or mecamylamine (10 μM) was introduced to the superfusion medium 5 min after S1. Direct stimulation of nicotinic receptors was assessed through the application of 1 μM nicotine (a nicotinic receptor agonist) following S1.

### 2.3 Incorporation of [ ^3^H] Choline

Segments prepared from frontal cortex, hippocampus, or striatum were incubated with [^3^H]choline to detect its incorporation. The sizes of segments were approximately 0.5 mm in length, 0.5 to 1 mm in width, and 0.3 mm in thickness. Three segments were set in each well of a non‐tissue culture‐treated plate (24 wells, flat bottom with low evaporation lid; Falcon, Corning Incorporated, USA), and incubated in 0.5 mL of isotonic phosphate buffer (137 mM NaCl, 2.7 mM KCl, 1.3 mM MgCl_2_, 1.5 mM CaCl_2_, 1.5 mM KH_2_PO_4_, 8.2 mM Na_2_HPO_4_, and 10 mM D‐(+)‐glucose (pH 7.4)). The isotonic phosphate buffer was oxygenated with a mixture of 95% O_2_ and 5% CO_2_ prior to use. Following a 15‐min pre‐incubation at 37°C, [^3^H]choline was added at a final concentration of 100 nM, and the mixture was immediately incubated. The incubation time was 15 min, during which the plate was constantly shaken at a speed of 60–100 cycles/min. Hemicholinium‐3 (HC‐3) was added upon preincubation. The incubation was stopped by cooling the plate in ice water; then an ACh esterase (AChE) inhibitor, “neostigmine” (final concentration of 30 μM), was immediately added into each well to suppress the AChE activity of the segments. After washing twice with aCSF containing 30 μM neostigmine, segments were picked up and transferred into a small eppendorf tube containing 0.5 mL of reverse‐phase high‐performance liquid chromatography (HPLC) elution solvent at 4°C. The composition of HPLC elution solvent was 0.04 M phosphate buffer (pH 4.6), methanol (3% vol), and 1 mM tetramethylammonium, in which 30 μM neostigmine was added. The eppendorf tubes containing the segments in HPLC elution solvent were incubated at 4°C overnight to extract the incorporated radioligands. The following day, the tubes were vortexed and then centrifuged at 4°C for 10 min at 10000 × *g* to separate the supernatant and precipitate. The supernatant was carefully aspirated and used for the measurement of total radioactivity and for HPLC analysis. The residual precipitate in the tube was dissolved in 0.2 mL of 0.3 M NaOH for use in protein assays. Because the protein level in the supernatant was negligible, the protein level in the precipitate was estimated as the total protein content of the incubated segments (Muramatsu et al. 2024a).

### 2.4 Measurement of CHT1 and Non‐CHT1 Activities

As mentioned above, the supernatants were extracted from the segments incubated with [^3^H]choline in both the absence and presence of 1 μM or 1 mM HC‐3. Given that HC‐3 at 1 μM selectively inhibits CHT1 (Muramatsu et al. 2019, 2022, 2024a), the difference in incorporated radioactivity between the absence and presence of 1 μM HC‐3 was designated as CHT1 activity. The differentiation in [^3^H] incorporation between the treatments with 1 μM and 1 mM HC‐3 was determined as non‐CHT1 activity.

### 2.5 Measurement of [ ^3^H]ACh Quantity

To estimate [^3^H]ACh formation from the incorporated [^3^H]choline, the supernatant extracted from the segments incubated with [^3^H]choline was analyzed using HPLC. The HPLC system used was LC‐10ADvp (Shimadzu Inc., Kyoto, Japan) with a reverse‐phase InertSustain C18 column (4.6 mm diameter ×250 mm, GL Sciences Inc., Tokyo, Japan). The flow rate of the HPLC elution solvent was 0.15 mL/min, and the effluent was collected in 1‐min fractions. The retention times of choline and ACh were determined using the radioactive standards [^3^H]choline and [^3^H]ACh as references, and the standards [^3^H]choline and [^3^H]ACh showed peaks at 23 and 27–28 min, respectively (Muramatsu et al. 2019, 2024a).

### 2.6 Radioligand Binding Experiments

Tissue homogenization commonly leads to receptor yield loss and alters receptor environments. To circumvent these artifacts and detect receptors in small tissues under natural conditions, we developed a tissue segment binding methodology (Muramatsu et al. 2005, 2024b). Radioligand binding experiments were conducted using mouse brain segments rather than homogenates. [^3^H]quinuclidinyl benzilate ([^3^H]QNB) was used as a radioligand to detect muscarinic receptors. Briefly, small segments prepared from frontal cortex, hippocampus, and striatum were incubated with [^3^H] QNB in the phosphate buffer at 20°C for 6 h, in the absence or presence of competitors. The segments were then washed with ice‐cold aCSF and solved in 0.3 M NaOH solution, as described in the [^3^H]choline‐uptake experiment. Nonspecific binding was determined by binding in the presence of 10 μM atropine. The nonspecific accumulation of radioligand in the interstitial space was removed during the washing procedure (Muramatsu et al. 2005, 2024b). The total binding density (Bmax) was estimated based on the specific binding of [^3^H]QNB at 10 nM (a saturated binding concentration). The proportions of M1 and M2 subtypes were estimated from the inhibition of 50 μM pirenzepine and 10 μM AF‐DX 116, respectively, at 2.5 nM [^3^H]QNB binding sites, according to our previous reports (Muramatsu et al. 2019, 2024b).

### 2.7 Statistical Analysis

**FIGURE 1:**
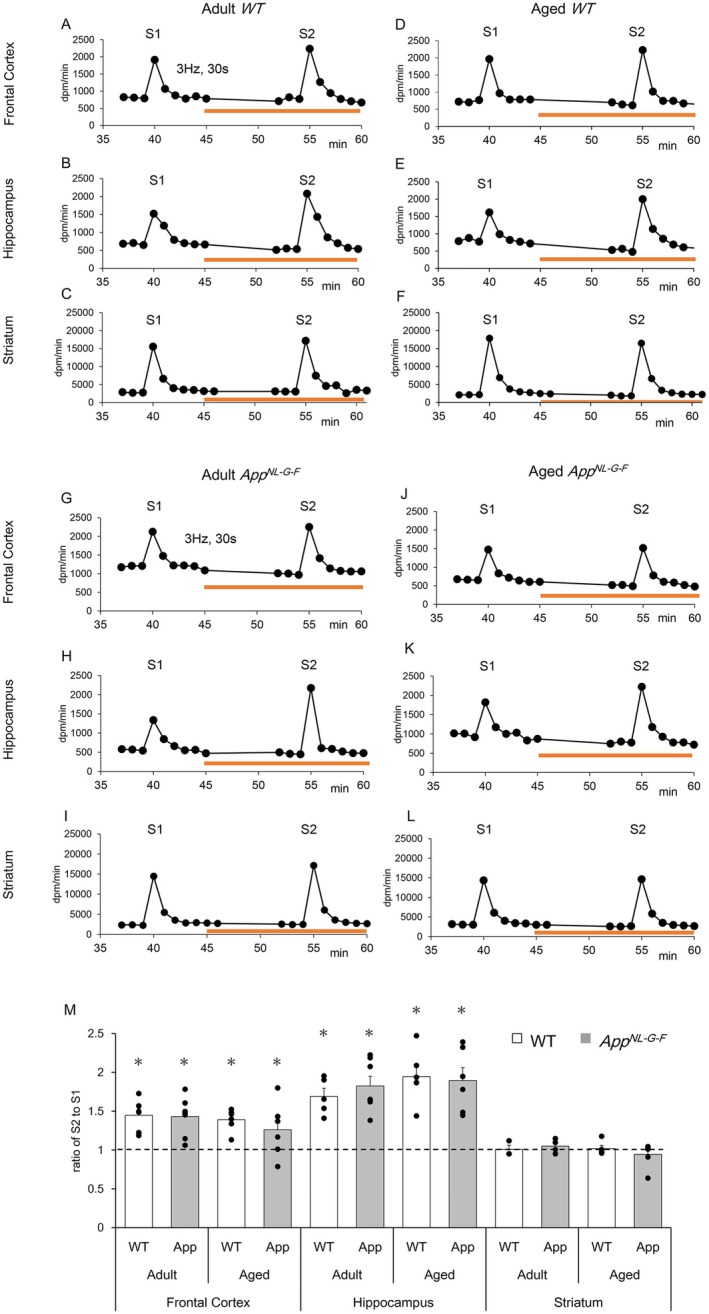
[^3^H]efflux evoked by electrical stimulation was enhanced by atropine (which inhibits presynaptic muscarinic feedback) in the frontal cortex and hippocampus of mice during superfusion experiments. Segments of the frontal cortex, hippocampus, and striatum isolated from wild type (WT) and Alzheimer's disease model mice (*App* ^*NL‐G‐F*^) mice were pre‐incubated with 0.1 μM [^3^H]choline for 15 min and then superfused. The superfusate was collected every minute. Electrical stimulation (at 3 Hz for 30 s) was delivered twice (S1 and S2) in each experiment. After the first stimulation (S1), 1 μM atropine was perfused (orange line). (A–C, G–I): Adult mice (6–8 months old). (D–F, J–L) Aged mice (12–17 months old). (A–F) WT mice. (G–L) *App* ^*NL‐G‐F*^ mice. The ordinate denotes [^3^H]efflux (dpm) in each fraction collected every minute. The abscissa shows the time after the commencement of superfusion. The [^3^H]efflux evoked under the treatment with 1 μM atropine (S2) was significantly enhanced in frontal cortex and hippocampus, but not in the striatum, at both adult and aged stages of WT and *App* ^*NL‐G‐F*^ mice. (M) The effects of 1 μM atropine on [^3^H]efflux evoked by electrical stimulation were summarized in three brain regions of adult and aged mice. Open columns: WT mice (WT). Gray columns: *App* ^*NL‐G‐F*^ mice (APP). The evoked [^3^H]efflux ratios at S2 compared to S1 were calculated and presented as the mean ± standard error of the mean (SEM) from 3 to 7 experiments (mice). The dots depict the individual values obtained from different mice. The asterisks (*) denote significant differences in the ratio of S2 to S1 (*p* < 0.05, Wilcoxson signed‐rank test).

**FIGURE 2:**
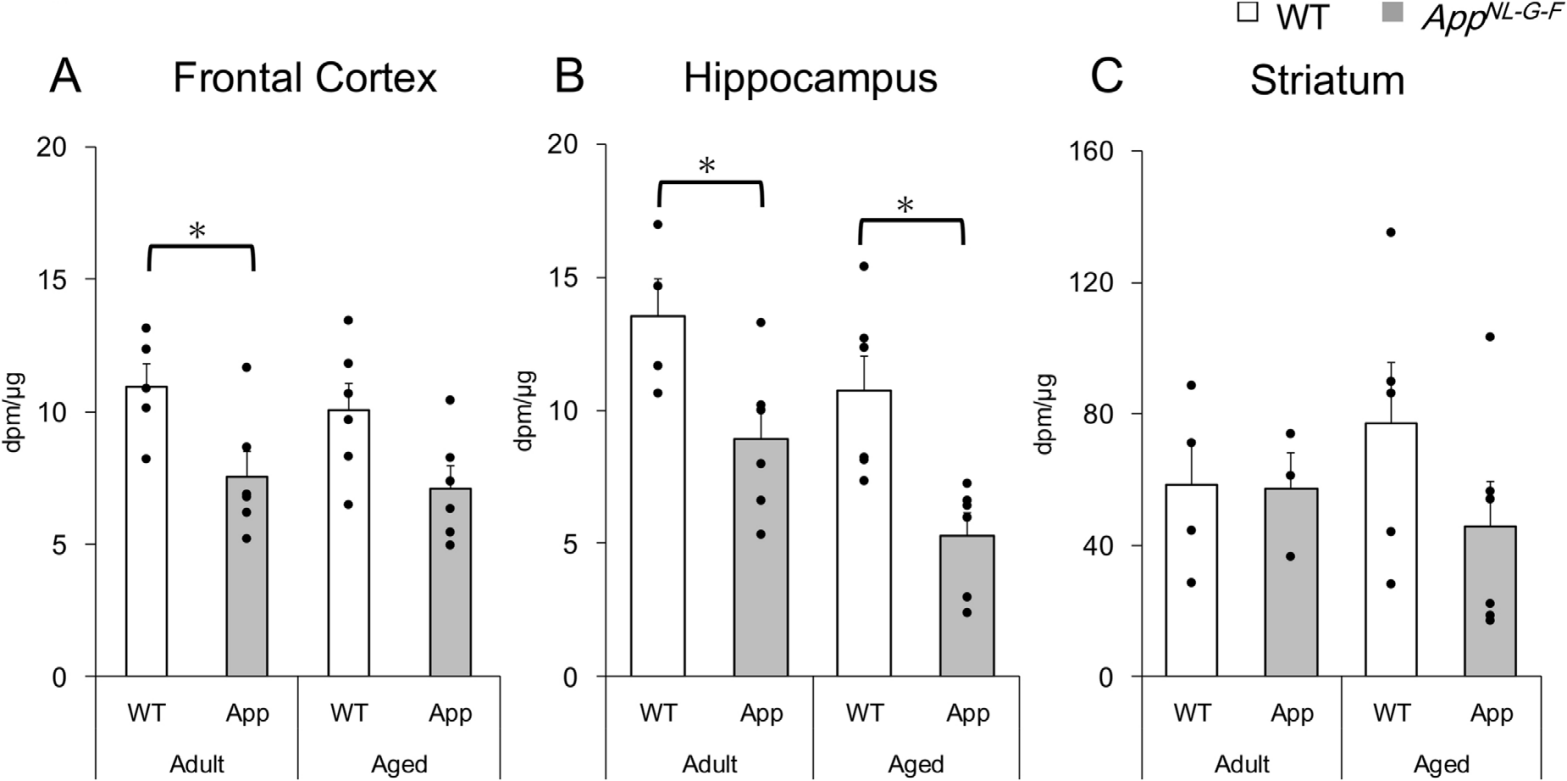
[^3^H]efflux evoked by electrical stimulation in *App* ^*NL‐G‐F*^ mice was reduced in frontal cortex (at adult) and hippocampus (in both adult and aged stages), compared to WT mice. The experimental conditions were the same as those shown in Figure 1. Net [^3^H]efflux evoked by electrical stimulation per segmental protein (dpm/μg) in the presence of 1 μM atropine was calculated. Open columns: WT mice. Gray columns: *App* ^*NL‐G‐F*^ mice (App). (A) Frontal cortex. (B) Hippocampus. (C) Striatum. Data are presented as mean ± SEM of 4–6 experiments (mice) and the dots depict the individual values obtained from different mice. The asterisks (*) denote significant differences between WT and *App* ^*NL‐G‐F*^ mice (*p* < 0.05, two‐way ANOVA with Tukey's post hoc test).

**FIGURE 3:**
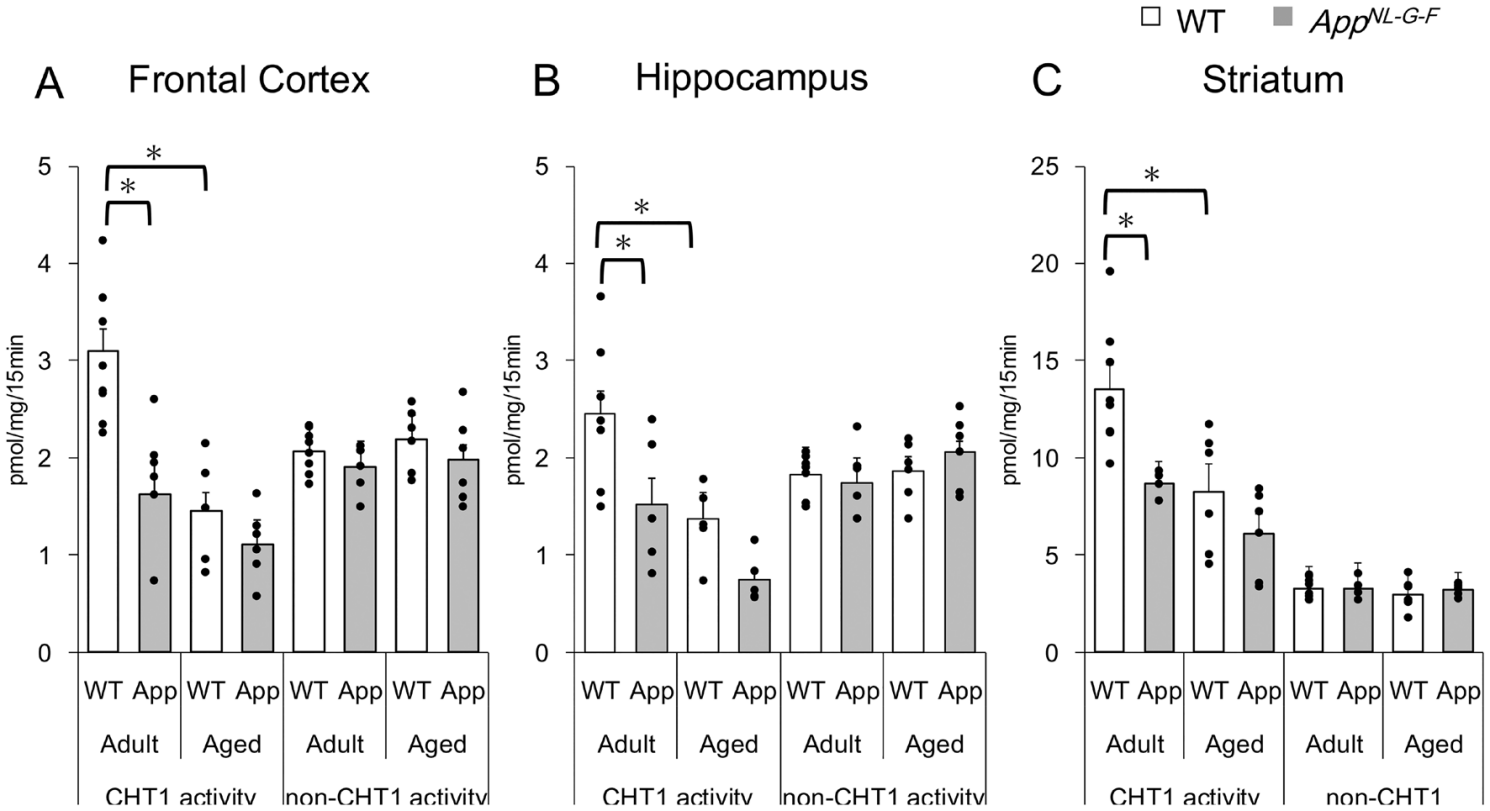
CHT1‐mediated [^3^H]choline uptake decreased with aging and in *App* ^*NL‐G‐F*^ mice. Brain segments were incubated with 0.1 μM [^3^H]choline for 15 min in the absence and presence of 1 μM or 1 mM HC‐3. CHT1 activity was defined as the uptake inhibited by 1 μM HC‐3, and a further inhibition by 1 mM HC‐3 was estimated as non‐CHT1 activity (see Methods and Results for further details). (A) frontal cortex; (B) hippocampus; (C) striatum. Open columns, WT mice; Gray columns, *App* ^*NL‐G‐F*^ mice (App). Each column shows mean ± SEM of 4–8 experiments (mice) and the dots depict the individual values obtained from different mice. The asterisks (*) denote significant differences between WT and *App* ^*NL‐G‐F*^ mice or between adult and aged WT mice (*p* < 0.05, two‐way ANOVA with Tukey's post hoc test).

**FIGURE 4:**
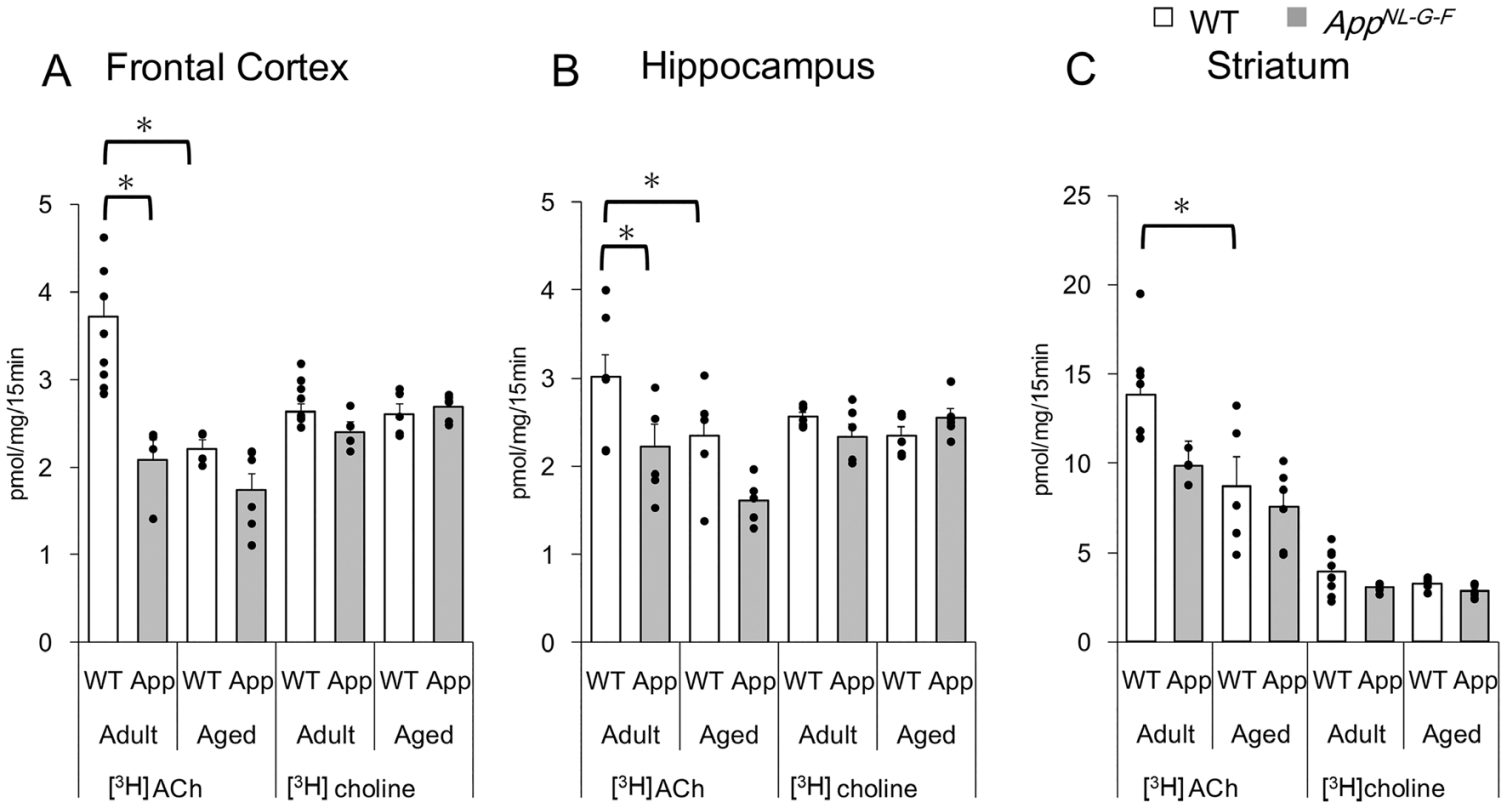
A reduction in the quantity of [^3^H]ACh is seen associated with aging and during the early stages in *App* ^*NL‐G‐F*^ mice. Brain segments were incubated with 0.1 μM [^3^H]choline for 15 min. [^3^H]ACh and [^3^H]choline that accumulated in the segments were extracted and analyzed with HPLC. (A) frontal cortex. (B) hippocampus. (C) striatum. Open columns, WT mice; Gray columns, *App* ^*NL‐G‐F*^ mice (App). Each column shows mean ± SEM of 4–8 experiments (mice) and the dots depict the individual values obtained from different mice. The asterisks (*) denote significant differences between WT and *App* ^*NL‐G‐F*^ mice or between adult and aged WT mice (*p* < 0.05, two‐way ANOVA with Tukey's post hoc test).

**FIGURE 5:**
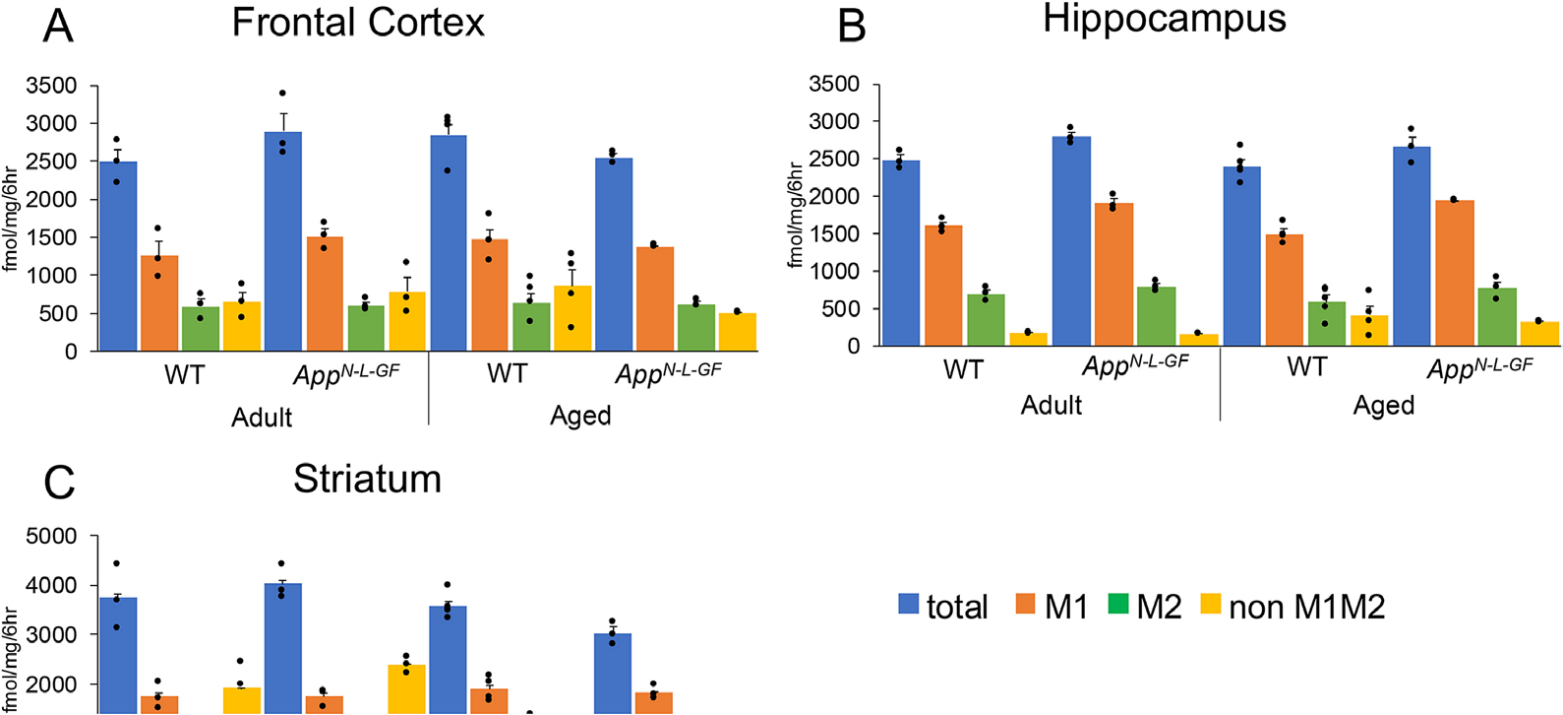
No significant changes are noted in the density and subtype proportions of muscarinic receptors across the three brain regions in both WT and *App* ^*NL‐G‐F*^ mice. [^3^H]QNB binding was performed in the frontal cortex, hippocampus, and striatum segments of mice. The total density was determined as the specific binding of 10 nM [^3^H]QNB. M1 and M2 subtypes were respectively extrapolated from the inhibition by 50 μM pirenzepine and 10 μM AF‐DX 116 at 2.5 nM [^3^H]QNB binding sites. The residual proportion was estimated as non‐M1/M2. (A) frontal cortex. (B) hippocampus. (C) striatum. Each column shows the mean ± SEM of 3–5 experiments and the dots depict the individual values obtained from different mice.

All data were presented as the mean ± standard error of the mean (SEM). Statistical analyses were performed with EZR version 1.61 (Saitama Medical Center, Jichi Medical University, Saitama, Japan), which is a graphical user interface for R version 4.2.3 (The R Foundation for Statistical Computing, Vienna, Austria) (Kanda 2013). Figure 1 was analyzed by using the Wilcoxon signed‐rank test, and Figures 2, 3, 4, 5 were analyzed by using two‐way ANOVA with Tukey's post hoc test, after the confirmation of normality of the data (Shapiro–Wilk test). No test for outliers was conducted on the data obtained in this study, and there were no predetermined exclusion criteria.

### 2.8 Reagents

Choline chloride [methyl‐^3^H] ([^3^H]choline, NET109, specific activity 80.8 Ci/mmol) and [^3^H]quinuclidinyl benzilate ([^3^H]QNB, NET656, specific activity 31.7 Ci/mmol) were purchased from PerkinElmer Inc. (Boston, MA, USA). HC‐3 (cat. no. H108), atropine sulfate (cat. no. A0257), pirenzepine dihydrochloride monohydrate (cat. no. P7412), AF‐DX 116 (11‐[[2[(Diethylamino)methyl]‐1‐piperidinyl]acetyl]‐5,1 1‐dihydro‐6H‐pyrido[2,3‐b][1,4]denzodiazepin‐6‐one, cat. no. AML0435), mecamylamine hydrochloride (cat. no. M9020) and nicotine tartrate dihydrate (cat. no. BP821) were acquired from Sigma‐Aldrich (St. Louis, MO, USA).

## 3 Results

### 3.1 [ ^3^H]ACh Release in Superfusion Experiments

An ultra‐mini superfusion system was used to monitor [^3^H]ACh release from cholinergic terminals/neurons in mouse brain segments. Figure 1A–F shows representative traces wherein the segments of frontal cortex, hippocampus, and striatum from WT mice were preloaded with [^3^H]choline and then superfused. Electrical stimulation at 3 Hz for 30 s evoked a transient elevation in [^3^H]efflux. The evoked [^3^H]efflux was completely inhibited by 0.5 μM tetrodotoxin (data not shown). Furthermore, HPLC analysis revealed a dominant proportion of [^3^H]ACh in the superfusate, which had been collected during electrical stimulation after treatment with ACh esterase inhibitor (Figure S1). Based on these results and our previous reports (Muramatsu et al. 2019; Arakawa et al. 2023), we concluded that the [^3^H]efflux evoked by electrical stimulation was cholinergic in origin and reflected [^3^H]ACh release.

ACh release is well‐established as being regulated by presynaptic cholinergic receptors. We first evaluated the presynaptic muscarinic receptor‐mediated feedback. Before atropine treatment, the quantities of evoked efflux (S1) were markedly low in the frontal cortex and hippocampus compared to those observed in the striatum. The net effluxes demonstrated reproducibility upon repeated stimulation within each experimental session when atropine was not treated. Therefore, the effects of atropine were simply compared between before and after treatment with atropine. The administration of 1 μM atropine following the first stimulation (S1) significantly enhanced the [^3^H]efflux evoked by the second stimulation (S2) in segments from frontal cortical and hippocampal regions of adult WT mice (6–8 months old) (1.44 ± 0.08 and 1.69 ± 0.10 folds relative to S1 in the frontal cortex (*p* = 0.02) and hippocampus (*p* = 0.03), respectively), whereas no enhancement was observed in striatal segments of adult WT mice (1.00 ± 0.05 folds to S1) (Figure 1A–C,M). A similar atropine‐induced enhancement was noted in the frontal cortex and hippocampal segments of aged WT mice (12–17 months old) (Figure 1D–F,M), indicating that presynaptic muscarinic receptor‐mediated feedback is functional in both adult and aged WT mice. Subsequently, we investigated the potential involvement of presynaptic nicotinic receptors. Nevertheless, mecamylamine (a nicotinic receptor antagonist, 10 μM) had no effect on the [^3^H]efflux induced by electrical stimulation, and nicotine itself (a nicotinic receptor agonist, 1 μM) did not influence either the basal [^3^H]overflow or the [^3^H]efflux evoked by electrical stimulation (data not shown). Consequently, we inferred that ACh release from cholinergic terminals in the frontal cortex and hippocampus is modulated via presynaptic muscarinic but not nicotinic receptors. Thus, presynaptic muscarinic receptor‐mediated feedback was exclusively examined in the following experiments.

To evaluate the presynaptic muscarinic receptor‐mediated feedback in AD mice, we performed superfusion experiments with brain segments obtained from *App* ^*NL‐G‐F*^ mice (Figure 1G–L). In alignment with WT mice, treatment with 1 μM atropine subsequent to S1 enhanced the [^3^H]efflux evoked by electrical stimulation (S2) in the frontal cortex and hippocampus of both adult and aged App^*NL‐G‐F*^ mice (1.43 ± 0.09 and 1.26 ± 0.1 folds to S1 in adult and aged frontal cortex (*p* = 0.01 and *p* = 0.04, respectively), and 1.82 ± 0.12 and 1.89 ± 0.16 folds to S1 in adult and aged hippocampus (*p* = 0.01 and *p* = 0.01, respectively)) (Figure 1G–M). These results suggested that [^3^H]ACh release was negatively regulated by the presynaptic muscarinic feedback system in the frontal cortex and hippocampus, regardless of Aβ_42_ overproduction. In contrast, atropine had a minimal effect on [^3^H]efflux in the striatum of WT and *App* ^*NL‐G‐F*^ mice, indicating that muscarinic auto‐regulation was less dominant in the striatal cholinergic neurons.

Next, [^3^H]ACh release was quantitatively evaluated. Here, net [^3^H]efflux under 1 μM atropine treatment was used to verify cholinergic activity without presynaptic modulation. The amounts of evoked [^3^H]efflux were 10.94 ± 0.79 dpm per μg of segmental protein in the frontal cortex and 13.50 ± 1.44 dpm/μg in the hippocampus of adult WT mice. The efflux exhibited a tendency of a gradual decline along aging, although no statistical differences were noted in the frontal cortex and hippocampus of WT mice (Figure 2A,B). In contrast, the amounts of evoked [^3^H]efflux in the frontal cortex (7.55 ± 0.94 dpm/μg) and hippocampus (8.93 ± 1.16 dpm/μg) of adult *App* ^*NL‐G‐F*^ mice were significantly lower than those in adult WT mice (F(1, 16) = 16.06, *p* = 0.04 in frontal cortex and F(1, 17) = 22.05, *p* = 0.03 in hippocampus, respectively). Further reduction was observed in the hippocampus of aged *App* ^*NL‐G‐F*^ mice (Figure 2B). [^3^H]efflux in the striatum was significantly higher than that in the frontal cortex and hippocampus and did not change with aging or between WT and AD mice (Figure 2C). These results suggested that ACh release from cholinergic terminals of the frontal cortex and hippocampus was specifically reduced at an early stage in *App* ^*NL‐G‐F*^ mice.

### 3.2 Effects on CHT1 and Non‐CHT1 Activities

To explore the mechanisms associated with the reduction in [^3^H]ACh release, we examined [^3^H]choline incorporation into cholinergic terminals/neurons through CHT1. CHT1 activity was measured as a difference in incorporation of [^3^H]choline in the absence versus presence of 1 μM HC‐3 (a selectively‐inhibiting concentration of CHT1) (Muramatsu et al. 2019). CHT1 activity in the frontal cortex and hippocampus of both WT and *App* ^*NL‐G‐F*^ mice was less than one‐fourth of that observed in the striatum of both adult and aged mice (see the different ordinate scales in Figure 3A–C), consistent with distinct densities of cholinergic innervation. CHT1 activities in three brain regions of adult *App* ^*NL‐G‐F*^ mice were significantly lower than those in adult WT mice (Figure 3A–C) (frontal cortex: 1.62 ± 0.27 in *App* ^*NL‐G‐F*^ mice vs. 3.09 ± 0.24 pmol/mg/15 min in WT mice, F(1, 21) = 11.02, *p* = 0.004; hippocampus: 1.51 ± 0.25 in *App* ^*NL‐G‐F*^ mice vs. 2.45 ± 0.28 pmol/mg/15 min in WT mice, F(1, 20) = 11.28, *p* = 0.04; striatum: 8.70 ± 1.31 in *App* ^*NL‐G‐F*^ mice vs. 13.54 ± 1.11 pmol/mg/15 min in WT mice, F(1, 20) = 9.57, *p* = 0.04). Furthermore, CHT1 activities in three brain regions were significantly reduced with aging in WT mice.

In contrast, non‐CHT1 activity, estimated as the difference in [^3^H]choline incorporation between the treatments with1 μM and 1 mM HC‐3, did not exhibit any difference between WT and *App* ^*NL‐G‐F*^ mice (Figure 3A–C), suggesting no change in the basal metabolism of brain segments comprising various cell types (Bazalakova and Blakely 2006). These findings strongly indicate a selective decline in CHT1 activity at an early stage in App^*NL‐G‐F*^ mice, diverging from the sustained decline observed with aging in WT mice.

### 3.3 Effects on [ ^3^H]ACh Formation/Storage

It is well known that choline incorporated through CHT1 is preferentially converted to ACh, which is stored in cholinergic vesicles (Haga 1971; Molenaar et al. 1973; Kuhar and Murrin 1978). Therefore, we measured the amounts of [^3^H]ACh and [^3^H]choline accumulated in the segments after incubation with [^3^H]choline. The quantity of [^3^H]ACh was approximately 2–4 pmol/mg/15 min in the frontal cortex and hippocampus of WT mice, while its quantity in the striatal content was observed to be fourfold greater (Figure 4). The quantities of [^3^H]ACh in WT mice were significantly reduced with aging (frontal cortex: 3.70 ± 0.23 in adult vs. 2.21 ± 0.09 pmol/mg/15 min in aged, F(1, 19) = 13.58, *p* = 0.002; hippocampus: 3.01 ± 0.25 in adult vs. 2.34 ± 0.27 pmol/mg/15 min in aged, F(1, 16) = 11.85, *p* = 0.03; striatum: 13.85 ± 0.98 in *App* ^*NL‐G‐F*^ mice vs. 8.73 ± 1.61 pmol/mg/15 min in aged, F(1, 18) = 11.84, *p* = 0.01). In the frontal cortex and hippocampus of *App* ^*NL‐G‐F*^ mice, the quantity of [^3^H]ACh in adult mice was significantly lower than that in age‐matched WT mice (Figure 4A,B) (frontal cortex: 2.07 ± 0.22 in *App* ^*NL‐G‐F*^ mice vs. 3.70 ± 0.23 pmol/mg/15 min in WT mice, F(1, 19) = 20.38, *p* = 0.0003; hippocampus: 2.22 ± 0.25 in *App* ^*NL‐G‐F*^ mice vs. 3.01 ± 0.25 pmol/mg/15 min in WT mice, F(1, 16) = 18.61, *p* = 0.009), with values exhibiting a diminishing trend with aging. The quantity of [^3^H]ACh in the striatum of *App* ^*NL‐G‐F*^ mice was slightly lower at adult age than in WT mice, although a statistical difference was not observed (Figure 4C). In contrast, [^3^H]choline levels remained unchanged with aging or Aβ overexpression (Figure 4A–C). These outcomes imply that [^3^H]ACh formation and storage may be predominantly and selectively compromised within the forebrain of *App* ^*NL‐G‐F*^ mice.

### 3.4 Effects on Muscarinic Receptors

Effects of aging and Aβ_42_ overproduction on muscarinic receptors were examined by the radioligand binding method. Here, tissue segments were used instead of whole tissue homogenates to maintain the receptor environment and avoid yield losses associated with homogenization (Muramatsu et al. 2024b). The muscarinic receptor density (Bmax), estimated from [^3^H]QNB binding at 10 nM (a saturated concentration), was 2489 ± 165 fmol/mg protein in the adult WT frontal cortex, 2479 ± 69 fmol/mg in the adult WT hippocampus, and 3738 ± 373 fmol/mg in the adult WT striatum, and there were no significant changes between adult and aged or between WT and *App* ^*NL‐G‐F*^ mice (Figure 5A–C). The M1‐subtype (pirenzepine‐sensitive sites) was the major subtype (50%–60% in proportion) in the three brain areas. The proportions of M1‐subtype, M2‐subtype (AF‐DX 116‐sensitive sites), and the remaining proportion (non‐M1, M2 sites) were not significantly altered by aging and Aβ_42_ overproduction.

## 4 Discussion

Utilizing the ultra‐mini superfusion methodology, we effectively detected [^3^H]ACh release from small segments of mouse brain (frontal cortex, hippocampus, and striatum) in response to electrical stimulation. The observed quantities of released [^3^H]ACh were considerably lower in the frontal cortex and hippocampus (less than one‐fourth) in comparison to striatal outputs. This distinction likely correlates with the varying cholinergic innervation densities across brain regions. The striatum contains a substantial population of cholinergic interneurons in addition to external innervation (Kljakic et al. 2017; Lim et al. 2014).

[^3^H]ACh release in the frontal cortex and hippocampal segments was significantly enhanced through atropine (a muscarinic antagonist); however, it remained unaffected by mecamylamine (a nicotinic antagonist), and the nicotinic receptor agonist nicotine failed to influence either the basal or stimulated [^3^H]efflux. Consequently, these results suggest that ACh release from cholinergic terminals within the frontal cortex and hippocampus is negatively autoregulated via presynaptic muscarinic but not nicotinic receptors (Zhang et al. 2002; Muramatsu et al. 2022). In contrast, dopaminergic terminals demonstrate that nicotine acts on presynaptic nicotinic receptors to elicit dopamine release (Garcao et al. 2013, 2014).

Under conditions where presynaptic feedback was inhibited, we observed a trend suggesting that ACh release from cholinergic terminals in the frontal cortex and hippocampus decreased progressively with aging in WT mice, indicating gradual age‐related degeneration. Conversely, significant reductions in ACh release were observed at an early age (adult; 6–8 months) in *App* ^*NL‐G‐F*^ mice, affirming that cholinergic dysfunction manifests at an early stage in these mice prior to age‐dependent degeneration.

Cholinergic dysfunction within the forebrain, particularly affecting the nucleus basalis of Meynert (NBM) and medial septal nucleus (MSN), has been extensively reported during the early clinical phases in patients with AD (Giacobini et al. 2022; Giovacchini et al. 2019). The severe loss of cholinergic innervation and neurons in the NBM and MSN becomes increasingly pronounced as clinical and pathological stages progress (Butler et al. 2018; Nell et al. 2015; Al‐Shaikh et al. 2020). The *App* ^*NL‐G‐F*^ mice begin to develop typical β‐amyloid plaques starting after 4 months of age, demonstrating synaptic alterations, as indicated by reductions in synaptophysin and PSD95 immunoreactivities (Saito et al. 2014). Thus, these model mice show significant inflammatory responses due to β‐amyloid accumulation (Saito et al. 2014; Sasaguri et al. 2022), leading to memory impairment without overt cholinergic neuronal loss in the NBM (Hirota et al. 2023). Our preliminary studies also confirmed the early‐stage presence of β‐amyloid plaques in *App* ^*NL‐G‐F*^ mice (Figure S2), with no quantitative changes detected for CHT1 and synaptophysin in tissue extracts (Figure S3). The reasons behind these discrepant results remain unclear, although differences between total amounts measured in tissue extracts and local levels detected in cholinergic terminals via immunohistochemistry may account for this inconsistency. Further meticulous exploration is warranted.

It is well established that choline uptake via CHT1 in cholinergic terminals is exquisitely coupled to ACh synthesis (Haga 1971; Molenaar et al. 1973; Kuhar and Murrin 1978). Given that a significant proportion of CHT1 is localized to cholinergic vesicles, the transporter must translocate to the plasma membrane to incorporate extracellular choline. Notably, the trafficking of the transporter is enhanced following exocytosis/depolarization, resulting in increased CHT1 proteins at the plasma membrane (Okuda et al. 2002; Ribeiro et al. 2003; Ferguson et al. 2003; Nakata et al. 2004). The presence of CHT1 at the plasma membrane is also augmented by behaviorally and pharmacologically induced activity of cholinergic neurons both in vivo and in vitro (Simon and Kuhr 1975; Simon et al. 1976; Murrin and Kuhar 1976; Ferguson et al. 1994, 2003). In this study, we observed a reduction in the quantity of [^3^H]ACh that coincided with decreased CHT1 activity in the *App* ^*NL‐G‐F*^ mouse model. This suggests that impaired choline metabolism may be associated with a decrease in CHT1 protein levels at the plasma membrane, which could indicate reduced cholinergic neuron activity in these mice in vivo. Previous research has shown that Aβ has direct inhibitory effects on both ACh synthesis and release (Kar et al. 1996; Pedersen et al. 1996). Notably, the current findings offer insights into the potential interaction between APP and CHT1, which may promote endocytosis of CHT1 from the cell surface (Wang et al. 2007; Cuddy et al. 2015), resulting in a diminished presence of CHT1 on the plasma membrane. Moreover, cholinergic dysfunction has been noted to occur before the formation of amyloid plaques in another AD mouse model (Tg2576) (Watanabe et al. 2009). Thus, it would be interesting to investigate the relationship between cellular localization and activity of CHT1 and Aβ pathology across different AD‐model mice.

In contrast to CHT1, non‐CHT1 activity showed no signs of alteration due to aging or Aβ overproduction. The non‐CHT1 activity, primarily mediated by a low‐affinity choline transporter, is associated with the basal metabolic processes of cells (Bazalakova and Blakely 2006). The absence of changes in non‐CHT1 activity suggests that the overall basal cellular metabolism within the brain regions examined remains largely unaffected by aging and/or Aβ overproduction. Correspondingly, we observed no changes in the density or subtype distribution of muscarinic receptors in radioligand binding experiments. These results suggest that the fundamental viability of brain regions containing various cell types is minimally impacted by aging and/or Aβ production.

In summary, our experiments employing ultra‐mini superfusion, choline incorporation, and radioligand receptor binding in small segments of mouse brain provided insight into the pharmacological properties at cholinergic synapses, encompassing ACh release, choline uptake, ACh/choline metabolism, and presynaptic feedback. A clear reduction in ACh release was evident in samples from the frontal cortex and hippocampus of *App* ^*NL‐G‐F*^ mice, closely associated with inhibited CHT1 activity and reduced ACh formation and storage in cholinergic terminals. Notably, there were no significant changes in basal cellular metabolism or muscarinic receptors. Thus, presynaptic cholinergic dysfunction appears to occur early and selectively in the forebrain of AD mice, with Aβ pathology linked to diminished CHT1 function, independently of age‐related degeneration.

## Author Contributions

**Itsumi Nagai‐Arakawa:** investigation, conceptualization, writing – original draft, formal analysis, data curation, methodology, validation, visualization, writing – review and editing. **Ikunobu Muramatsu:** conceptualization, methodology, data curation, investigation, validation, formal analysis, project administration, writing – original draft, visualization, funding acquisition, writing – review and editing. **Junsuke Uwada:** methodology, writing – review and editing, funding acquisition. **Yo Tsuda:** investigation, methodology, data curation, writing – review and editing. **Akinori Tokunaga:** methodology, investigation, writing – review and editing. **Ai Irie:** methodology, investigation, writing – review and editing. **Hideyuki Maeda:** methodology, investigation, writing – review and editing. **Yuta Madokoro:** data curation, methodology, writing – review and editing. **Toyohiro Sato:** data curation, methodology, writing – review and editing. **Yuto Uchida:** data curation, methodology, writing – review and editing. **Takashi Saito:** resources, writing – review and editing. **Takaomi C. Saido:** resources, writing – review and editing. **Kiyonao Sada:** supervision, writing – review and editing. **Takayoshi Masuoka:** methodology, project administration, writing – review and editing, funding acquisition. **Noriyuki Matsukawa:** conceptualization, methodology, supervision, project administration, writing – review and editing, writing – original draft, funding acquisition.

## Conflicts of Interest

The authors declare no conflicts of interest.

### Peer Review

The peer review history for this article is available at https://www.webofscience.com/api/gateway/wos/peer‐review/10.1111/jnc.70081.

## Supporting information


Data S1.

## Data Availability

The data that support the findings of this study are available from the first author corresponding authors upon reasonable request.
