# Supplementary material for: Evaluation of the Alterations in Central Cholinergic Neurotransmission in Aging and Amyloid Precursor Protein Knock‐In Mice
Source: J Neurochem. 2025 May 13;169(5):e70081. doi: 10.1111/jnc.70081 (PMC12070134; doi:10.1111/jnc.70081)
Supplement: Supplementary file 1 — Data S1. [file JNC-169-0-s001.pdf]

Supplementary Materials

Evaluation of the alterations in central cholinergic neurotransmission in aging and amyloid precursor protein knock-in mice

Itsumi Nagai-Arakawa[a,b], Ikunobu Muramatsu[b,c*], Junsuke Uwada[c], Yo Tsuda[a], Akinori Tokunaga[d], Ai Irie[d], Hideyuki Maeda[d], Yuta Madokoro[a], Toyohiro Sato[a], Yuto Uchida[a], Takashi Saito[e], Takaomi C Saido[f], Kiyonao Sada[b], Takayoshi Masuoka[c], Noriyuki Matsukawa[a*]

[a] Department of Neurology, Nagoya City University Graduate School of Medicine, 1 Kawasumi, Mizuho-ku, Nagoya, Aichi 467-8602, Japan

[b] Department of Genomic Science and Microbiology, Faculty of Medical Sciences, University of Fukui, Eiheiji, Fukui 910-1193, Japan

[c] Department of Pharmacology, School of Medicine, Kanazawa Medical University, 1-1 Daigaku, Uchinada, Ishikawa 920-0293, Japan

[d] Division of Laboratory Animal Resources, Life Science Support Center, University of Fukui, Eiheiji, Fukui 910-1193, Japan

[e] Department of Neurocognitive Science, Institute of Brain Science, Nagoya City University Graduate School of Medical Sciences, Nagoya, Aichi 467-8601, Japan

[f] Laboratory for Proteolytic Neuroscience, RIKEN Center for Brain Science, Wako, Saitama 351-0198, Japan

Co-correspondence: Noriyuki Matsukawa, Department of Neurology, Nagoya City University Graduate School of Medicine, 1 Kawasumi, Mizuho-ku, Nagoya, Aichi 467-8602, Japan

E-mail: norim@med.nagoya-cu.ac.jp

Tel. +81-52-853-8094; Fax: +81-52-852-3590

Co-correspondence: Ikunobu Muramatsu, Department of Pharmacology, School of Medicine, Kanazawa Medical University, 1-1 Daigaku, Uchinada, Ishikawa, 920-0293, Japan

Email: muramatu@u-fukui.ac.jp

Tel: +81-76-218-8107; Fax: +81-76-286-8191

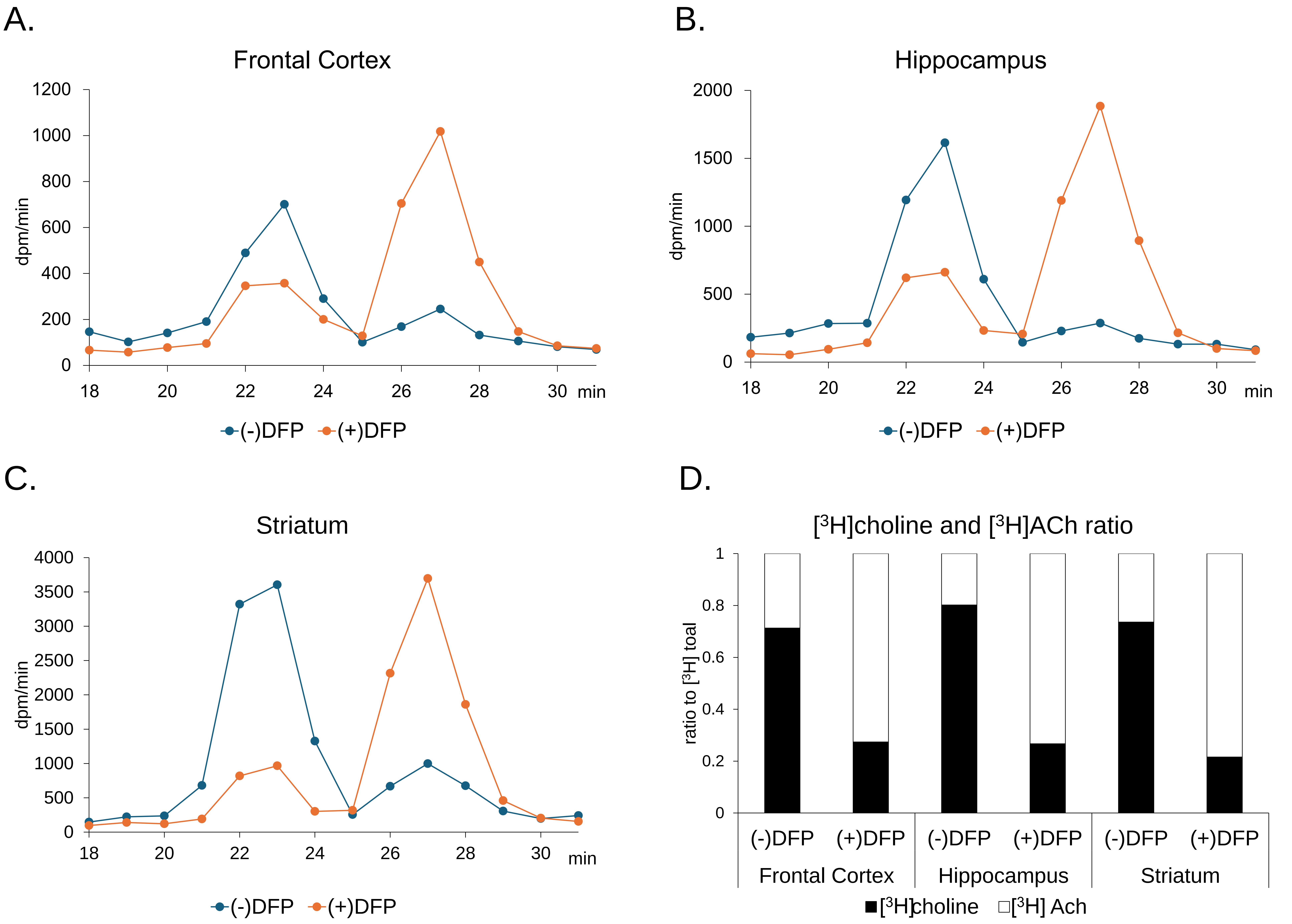

Supplementary Figure 1: Identification of $[^{3}H]$choline and $[^{3}H]$ACh components in the superfusate by HPLC analysis.

The proportion of $[^{3}H]$ACh was increased in the superfusate collected during electrical stimulation under treatment with diisopropylfluorophosphate (DFP, an ACh esterase inhibitor), suggesting that the evoked $[^{3}H]$efflux reflects $[^{3}H]$ACh release from cholinergic terminals.

All superfusion experiments were performed in the presence of 1 μM atropine to suppress pre-synaptic feedback. The segments were electrically stimulated twice at 20 min interval before and after treatment with 300 μM DFP in each experiment. Superfusates were collected for 60 s during stimulation (at 3 Hz, for 60 s) and then applied to HPLC analysis. **A, B, C:** The superfusates collected in the absence and presence of DFP were analyzed by HPLC and the elusion patterns were superimposed. Blue circles: before DFP treatment. Orange circles: during DFP treatment. Two distinct peaks were observed at 23 and 27–28 min retention time (abscissa), which were consistent with those of two standard radiolabeled compounds, $[^{3}H]$choline and $[^{3}H]$ACh, respectively (Muramatsu et al., 2019). **D:** Proportions of $[^{3}H]$choline (black parts) and $[^{3}H]$ACh (white parts) in the supefusates before and after DFP treatment. Mean of 3 experiments in WT mice.

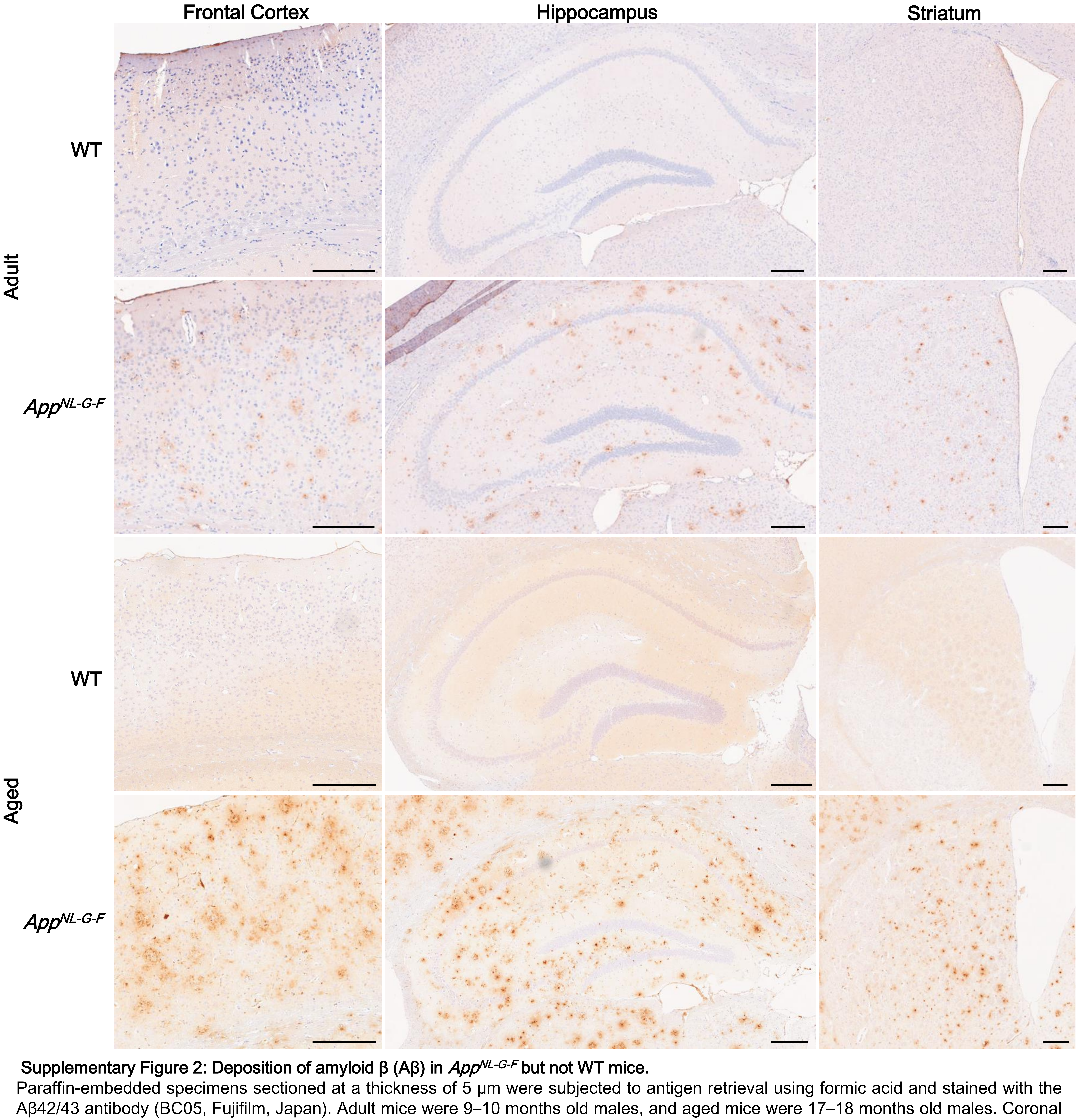

Supplementary Figure 2: Deposition of amyloid β (Aβ) in *App*$^{NL\text{-}G\text{-}F}$ but not WT mice.

Paraffin-embedded specimens sectioned at a thickness of 5 μm were subjected to antigen retrieval using formic acid and stained with the Aβ42/43 antibody (BC05, Fujifilm, Japan). Adult mice were 9–10 months old males, and aged mice were 17–18 months old males. Coronal sections at Bregma-2 mm were used for the frontal cortex and hippocampus, and coronal sections at Bregma +1 mm were used for the striatum. No Aβ deposition was observed in WT mice, regardless of age (Adult or Aged). In *App*$^{NL\text{-}G\text{-}F}$ mice, a small number of plaques were observed in Adult, and while numerous plaques were observed in aged mice. scale bar = 200 μm.

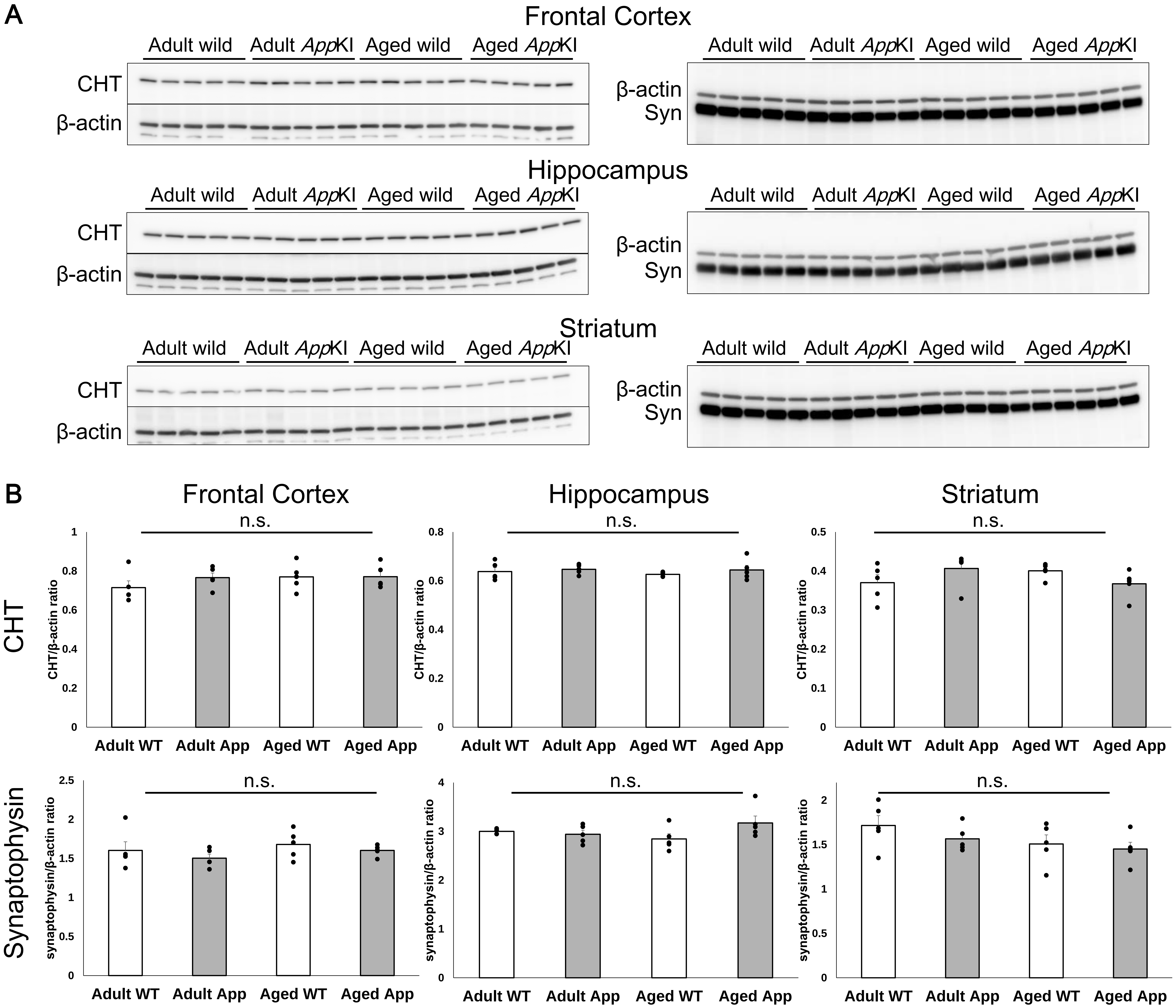

Supplementary Figure 3. Quantitative evaluation of CHT and synaptophysin (Syn).
Western blot was performed using extracts from the frontal cortex, hippocampus, and striatum. Neither CHT nor Syn showed significant differences in any of the regions examined.(n=5, two-way ANOVA; frontal cortex: CHT age ($F(1, 16)= 1.04$, $p=0.323$), genotype ($F(1, 16)=0.80$, $p=0.383$), interaction ($F(1,16)=0.75$, $p=0.399$), Syn age ($F(1, 16)= 1.39$, $p=0.256$), genotype ($F(1, 16)=1.40$, $p=0.255$), interaction ($F(1,16)=0.02$, $p=0.883$); hippocampus: CHT age ($F(1, 16)= 0.27$, $p=0.609$), genotype ($F(1, 16)=1.06$, $p=0.318$), interaction ($F(1,16)=0.11$, $p=0.748$), Syn age ($F(1, 16)= 0.14$, $p=0.710$), genotype ($F(1, 16)=1.82$, $p=0.196$), interaction ($F(1,16)=3.79$, $p=0.069$); striatum: CHT age ($F(1, 16)= 0.072$, $p=0.792$), genotype ($F(1, 16)=0.010$, $p=0.924$), interaction ($F(1,16)=4.37$, $p=0.053$), Syn age ($F(1, 16)= 3.15$, $p=0.095$), genotype ($F(1, 16)=1.29$, $p=0.272$), interaction ($F(1,16)=0.26$, $p=0.619$). Adult mice were 9–10 months old males, and aged mice were 15–16 months old males. All error bars represent standard errors.

			Frontal Cortex	Hippocampus	Striatum
**Figure 1**	Adult	WT	6 (5,1)	5 (5,0)	3 (3,0)
		App$^{NL\text{-}G\text{-}F}$	7 (5,2)	7 (5,2)	4 (2,2)
	Aged	WT	6 (6,0)	6 (6,0)	5 (5,0)
		App$^{NL\text{-}G\text{-}F}$	6 (6,0)	6 (6,0)	6 (6,0)
**Figure 2**	Adult	WT	5 (4,1)	4 (4,0)	5 (4,1)
		App$^{NL\text{-}G\text{-}F}$	6 (4,2)	6 (6,0)	6 (4,2)
	Aged	WT	6 (6,0)	6 (6,0)	6 (6,0)
		App$^{NL\text{-}G\text{-}F}$	6 (6,0)	6 (6,0)	6 (6,0)
**Figure 3**	Adult	WT	8 (7,1)	7 (6,1)	8 (7,1)
		App$^{NL\text{-}G\text{-}F}$	6 (4,2)	5 (4,1)	4 (4,0)
	Aged	WT	5 (5,0)	5 (5,0)	4 (4,0)
		App$^{NL\text{-}G\text{-}F}$	5 (5,0)	5 (5,0)	6 (6,0)
**Figure 4**	Adult	WT	8 (7,1)	7 (6,1)	8 (7,1)
		App$^{NL\text{-}G\text{-}F}$	6 (4,2)	6 (4,2)	4 (4,0)
	Aged	WT	5 (5,0)	5 (5,0)	4 (4,0)
		App$^{NL\text{-}G\text{-}F}$	6 (6,0)	5 (5,0)	6 (6,0)
**Figure 5**	Adult	WT	3 (2,1)	3 (2,1)	3 (2,1)
		App$^{NL\text{-}G\text{-}F}$	3 (3,0)	3 (3,0)	3 (3,0)
	Aged	WT	5 (3,2)	5 (3,2)	5 (3,2)
		App$^{NL\text{-}G\text{-}F}$	3 (2,1)	3 (2,1)	3 (2,1)

Supplementary Table 1: Numbers of mice used in the present study.
Numbers represent the numbers of total and (male, female) mice used in each figure.
